# Functional and Visual Outcomes of Orbital Floor Fracture Repair Using Titanium Mesh: A Retrospective Cross-Sectional Study

**DOI:** 10.7759/cureus.89168

**Published:** 2025-07-31

**Authors:** Sana Wazir, Nuzhat Rahil, Waseem Abbas, Suhair Shah, Ashwina Rahil

**Affiliations:** 1 Oral and Maxillofacial Surgery, Medical Teaching Institute, Lady Reading Hospital, Peshawar, PAK; 2 Ophthalmology, Medical Teaching Institute, Lady Reading Hospital, Peshawar, PAK; 3 Operative Dentistry, KRL (Khan Research Laboratories) Hospital, Islamabad, PAK

**Keywords:** diplopia, enophthalmos, extraocular motility, orbital floor fracture, titanium mesh, visual acuity

## Abstract

Objective

This retrospective study aims to evaluate the functional and visual outcomes of patients undergoing orbital floor fracture repair using titanium mesh implants.

Methods

Medical records of patients treated over four years for isolated or combined orbital floor fractures using titanium mesh were reviewed. Clinical outcomes assessed included diplopia, enophthalmos, ocular motility, and visual acuity, both pre- and postoperatively. Radiological evaluations were used to confirm anatomical restoration.

Results

A total of 25 patients (all males, mean age: 39.32 years) were included. Postoperative assessment showed significant improvement in diplopia in 72% (n = 18) and ocular motility in 68% (n = 17) of cases. Enophthalmos correction was achieved in 76% (n = 19) of patients. Visual acuity remained stable or improved in the majority of cases. Minor complications in the form of paresthesia were noted in 8% (n = 2) of patients, and visual complications were noted in 4% (n = 1). No major implant-related issues or infections were observed. Only 16% (n = 4) of patients required secondary intervention.

Conclusion

Titanium mesh is a reliable and effective material for repairing orbital floor fractures, offering favorable functional and visual outcomes with a low rate of complications. Its use facilitates accurate anatomical reconstruction and contributes to long-term orbital stability in adults.

## Introduction

Fracture of the bony orbit is a common finding occurring as a result of maxillofacial injuries. The treatment of orbital fractures can be quite challenging [1]. Orbital fractures comprise a total of 40% of craniofacial injuries [2]. The floor is the thinnest among the four walls of the orbit and is therefore more prone to fracture in any traumatic event [3]. Most surgeons agree that orbital fractures treated within the first two weeks of injury have the best clinical and functional outcomes [4].

Diplopia and restricted eye movements are common symptoms after orbital trauma, requiring surgical treatment of floor fractures. The main aim of surgery is to improve diplopia, though it may persist after the procedure. Diplopia can resolve, persist (improve, remain stable, or worsen), or be induced after repair of orbital floor injury [5]. From the literature, it is evident that postoperative diplopia persists in 52% of the patients. This number depends on several factors, including the implant material being used, the type of surgical method, the extent and severity of the fracture, and, most importantly, the timing of surgery [6].

Materials used for orbital floor fracture repair are autogenous (split cranial bone, cartilage, bone fragment, dermal fat, and rib), allogenic (human dura matter, lyophilized cartilage, banked bone, fascia lata, and heterogenic bovine bone graft), and alloplastic (silastic tantalum, stainless steel, vitallium, titanium, polymethylmethacrylate, polyvinyl sponge, polyurethane, polyethylene, Teflon, hydroxyapatite, gel foam, gel film, and supramid) [7]. With recent advances, there is a shift in trend from autologous bone grafts to alloplastic material to reduce donor site morbidity. The option for reconstruction techniques is multifactorial, depending upon the surgeon's preference, experience, and defect size of the fracture [8].

This retrospective study will allow analysis of real-world clinical outcomes over time to identify the trends and complications. It will correlate the anatomical repair with the patient-centered outcomes and assess the burden of orbital floor fractures on the resources of the institution. This study will also assess the long-term impact on the visual and functional outcomes, which is still under debate. There are gaps in the literature, with very few studies comprehensively analyzing long-term functional and visual outcomes with titanium mesh repair. The purpose of this study is to assess functional and clinical outcomes in the form of postoperative diplopia, ocular motility, enophthalmos, and visual acuity after repair of the orbital floor fracture using titanium mesh.

## Materials and methods

This retrospective study was conducted on the data of 25 patients with isolated or combined orbital floor fractures who underwent floor repair using titanium mesh at the Oral and Maxillofacial and Ophthalmology Unit of Lady Reading Hospital, Peshawar, Pakistan, from 2020 to 2024. All the patients who had orbital floor fractures due to road traffic accidents, sports injuries, and assaults, regardless of gender, ethnicity, and geographical background, were included in this study. Patients with orbital floor fractures due to firearm injuries, those below 16 years of age, those with incomplete medical records, and those who were lost to follow-up were excluded from this study.

After obtaining ethical approval from the Lady Reading Hospital, Medical Teaching Institute, Peshawar, Pakistan (Ref. No.: 235/LRH/MTI), and informed consent from the patients, the medical records from Health Management Information System (HMIS) were reviewed to extract demographic data, including age, gender, cause of orbital floor fracture, preoperative and postoperative records of visual acuity, diplopia, and amount of enophthalmos. Patients were diagnosed on clinical examination and radiological investigation. Clinical diagnosis of visual acuity was done using a Snellen chart; diplopia and ocular movement were checked by moving an index finger at one arm’s length in nine cardinal positions of gaze, confirmed by a force duction test for restricted eye movement; and enophthalmos was measured using a Hertel exophthalmometer. Hess charting was done to confirm diplopia. For radiological diagnosis, coronal and sagittal CT scans with 3D reconstructions were done. The location, size, and any associated injuries were also confirmed by CT.

Titanium implants from Shanghai Haosun Medical Instruments, Shanghai, China, were used, which were available in different sizes, that is, small, medium, and large, depending on the size of the defect. The thickness of the titanium mesh used was 0.1-0.3 mm, which is easy to shape and is lightweight.

All surgeries were performed by a maxillofacial surgeon under general anesthesia. The surgical approach used for orbital floor exploration was a transconjunctival approach with lateral canthotomy, infraorbital rim approach, and lower blepharoplasty. After exposing the orbital rim and floor, orbital floor exploration was performed, and retrieval of herniated content, that is, orbital fat and muscle, was done. A floor with a defect size measuring greater than 2 cm [2] was considered for reconstruction using titanium mesh, placed on sound bone posterolaterally and medially. Anteriorly, the mesh was secured using 6-mm microscrews along the infraorbital rim. Any associated fractures, if present, were treated accordingly.

Postoperatively, all patients received broad-spectrum intravenous antibiotics and anti-inflammatory medication in the form of intravenous steroid, that is, intravenous dexamethasone 8 mg once daily, along with pain control.

Postoperative outcomes, including diplopia, ocular motility, enophthalmos, and visual acuity, were assessed on the second postoperative day, at six weeks, and at six months. The medical records were also evaluated for complications, including infection, paresthesia, visual complications, and need for re-surgery, and the findings were reported in the results.

Data analysis

Statistical analysis for this cross-sectional study was performed using SPSS version 25 (IBM Corp., Armonk, NY). The demographic data, such as age and gender, were analyzed using frequencies and percentages. For scale variables, such as visual acuity, the mean and standard deviation were calculated. A paired t-test was performed for preoperative and postoperative visual acuity. For categorical variables, percentages were calculated, and a statistical comparison of preoperative variables was done with postoperative results. The study followed the STROBE (Strengthening the Reporting of Observational Studies in Epidemiology) guidelines for reporting cross-sectional studies.

## Results

This study was conducted in a tertiary care hospital in the maxillofacial and ophthalmology units of Medical Teaching Institute (MTI), Lady Reading Hospital (LRH), Peshawar, Pakistan. A total of 25 patients with orbital floor fractures were included during the study period (2020-2024). All patients were male, with ages ranging from 17 to 68 years (mean age: 39.32 ± 14.67 years).

**Figure 1:**
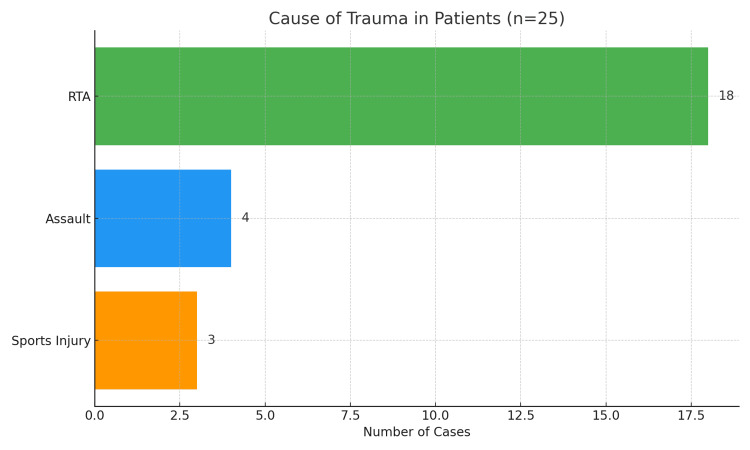
Causes of trauma in patients The horizontal bars illustrate the causes of trauma. RTA was the leading cause (n = 18, 72%), followed by assault (n = 4, 16%), and sports injury (n = 3, 12%). RTA is represented by green, assault by blue, and sports injuries by orange. RTA: Road traffic accidents.

In 92% (n = 23) of patients, there were associated injuries in the form of ocular, zygomaticomaxillary complex, and other facial bone fractures. The most common cause of trauma was road traffic accidents (RTA), as shown in Figure 1.

The visual acuity (VA) recorded on the Snellen chart on arrival was perception of light (PL) to 6/60 in 60% (n = 15) of patients, while 40% (n = 10) had VA of 6/60 or better. Preoperative vertical diplopia was present in all patients on arrival, while 72% (n = 18) of patients had enophthalmos of more than 2 mm recorded on the Hertel exophthalmometer. All the patients had restricted eye movement in up-and-down gaze on arrival, which was the indication after investigation for orbital exploration and reconstruction.

While analyzing the outcome variables on the first postoperative examination, 52% (n = 13) of patients did not show any improvements in diplopia, 88% (n = 22) had restricted ocular movements, and 76% (n = 19) had enophthalmos due to the surgically induced trauma and edema. However, the same outcomes had different results on further examination, that is, at the sixth week and sixth-month follow-up, as shown in Table 1.

**Table 1:** Outcomes Postoperative outcomes were assessed at the first postoperative visit, at six weeks, and at six months. The values are presented as n for numbers and % for percentages.

Outcomes	Examination at first postop visit, n (%)	Examination at 6 weeks, n (%)	Examination at 6 months, n (%)
Visual acuity			
PL to 6/60	15 (60%)	7 (28%)	05 (20%)
6/60 to better	10 (40%)	18 (72%)	20 (80%)
Diplopia			
Improved	12 (48%)	18 (72%)	18 (72%)
Not improved	13 (52%)	07 (28%)	07 (28%)
Enophthalmos			
Improved	06 (24%)	17 (68%)	19 (76%)
Not improved	19 (76%)	08 (32%)	06 (24%)
Ocular movements			
Restricted	22 (88%)	17 (68%)	08 (32%)
Not restricted	03 (12%)	08 (32%)	17 (68%)

While assessing the secondary outcomes, that is, complications, re-surgery was planned in 16% (n = 4) of patients, paresthesia was present in 8% (n = 2), and ocular complications were present in 4% (n = 1) of patients.

In inferential statistical analysis, a paired t-test was applied to continuous outcomes such as preoperative VA and VA at six months. There was a statistically significant improvement in VA at six months after surgery compared to the preoperative condition (p = 0.002). The confidence interval supports this improvement, as it does not include zero. Statistical significance was set at p < 0.05, as shown in Table 2.

**Table 2:** Paired t-test Comparison of preoperative and six-month postoperative VA was done using a paired t-test. Statistical significance was defined as a p-value < 0.05 (0.002), with the significant p-value denoted by an asterisk (*). The effect size (mean difference = –0.4) suggests a noticeable clinical improvement. VA: Visual acuity.

Pair	Mean	Std. deviation	Std. error mean	95% CI lower	95% CI upper	t	df	Sig. (two-tailed)
Preoperative VA vs postoperative VA at 6 months	-0.40000	0.57735	0.11547	-0.63832	-0.16168	-3.464	24	0.002^*^

## Discussion

Orbital floor fracture is one of the most common outcomes of trauma because of its anatomical morphology. Also, in a country like Pakistan, where RTA, assaults, and sports injuries are considered the leading factors contributing to these fractures. The diverse treatment modalities available for the repair of orbital floor fractures have different visual and functional outcomes. In our setup, the most common surgical management is placing a titanium mesh after releasing soft tissue herniation. This retrospective cross-sectional study analyzed the medical records of 25 patients with orbital floor fractures, all treated with titanium mesh for orbital reconstruction, to evaluate the functional and visual outcomes.

Orbital floor fractures are most common in young male adults. The most common causes are RTAs, fights, and sports accidents [9]. Similar results were found in our study, with all patients being males and the most common cause being RTAs, followed by assault and sports injury. In this part of the world, the majority of the male population are breadwinners and are involved in outdoor activities, while females are confined to their homes due to cultural dynamics. However, another study contradicts our findings because 28% of female patients presented with orbital floor fractures [10].

The mean age in our study was 39.32 years with a standard deviation (SD) of 14.67, a minimum age of 17 years, and a maximum age of 68 years. Similar results were observed in a scoping review on trauma care in low- and middle-income countries (LMICs) by Elwell et al., where the mean age was 30.8 years, and 74.3% of the male population in LMICs were affected [11]. The same study concluded that 60.4% of patients had an RTA as the leading cause, followed by assault and fall [11].

In the past decades, different treatment options for reconstruction of the orbital floor were present, including autogenous, allogenic, and alloplastic materials [7]. These reconstructive materials were not without any adverse effects and complications. In the early days, titanium mesh was only used for small orbital defects, but with advancements in research and clinical studies, the use of titanium mesh to cover larger defects has been proven [12]. Due to the widespread availability and acceptability of titanium mesh, in this study, we assessed the functional and visual outcomes of orbital reconstruction using titanium mesh.

In our study, the presenting complaint was preoperative diplopia with dimness of vision, restricted eye movements, and enophthalmos. In another study, the presenting complaint was edema, 80% of patients had diplopia, and 40% had restricted eye movement [13]. In contrast, we concluded that all patients in our study presented with diplopia and restricted eye movement, and 72% had enophthalmos.

Associated injuries were present in 92% (n = 23) of the patients in our study. However, a study conducted in Frankfurt concluded that only 1.8% of patients present with associated polytrauma [10]. The visual outcomes on the first postoperative examination were not very promising, except that no difference was recorded between pre- and first postoperative VA. The reason behind the lack of improvement in other visual outcomes was post-surgical trauma, edema, pain, and ocular injuries. The VA in our study improved four to five lines on the Snellen chart in 80% (n = 20) of patients by six months. In the remaining 20%, the VA was not improved. In contrast, a study done by Kansakar and Sundar reported traumatic blindness in 2.08% of patients [14].

In this study, diplopia recorded at six weeks and six months showed major improvement in 72% (n = 18) of patients, with no improvement in 28% (n = 7). A meta-analysis documented that 11% of patients had postoperative diplopia [15]. Another study revealed that postoperative diplopia improved in 75% of patients after management of orbital floor fracture, while it worsened in 10.7% of patients [5].

One study reported that 76% had restricted ocular motility preoperatively [6], whereas in our study, 100% (n = 25) of patients showed some type of restricted ocular movements on initial presentation. After floor repair, we found that ocular motility improved in 32% (n = 8) of patients at six weeks postoperatively and in 68% (n = 17) at six months. However, in a systematic review, no conclusive evidence was established between orbital floor fracture repair and eye movement [16].

We observed that the frequency of preoperative enophthalmos was 72% (n = 18) in this study. Another study revealed that the overall incidence of preoperative enophthalmos was 56.9%, which improved over time in 76.2% of patients [17]. This is comparable to our study, where postoperative enophthalmos improved at six weeks in 68% (n = 17) and at six months in 76% (n = 19) of patients.

In this study, re-surgery was planned for 16% (n = 4) of patients, paresthesia was reported in 8% (n = 2), and no infections were documented related to titanium mesh. In one of the local studies conducted in a tertiary care hospital, the postoperative infection rate was 4.5%, and paresthesia was present in 2.5% of patients [18].

 The limitation of this study is the small sample size and a deficient comparison with other reconstructive materials used for orbital floor repair. Therefore, more prospective research with a bigger sample size is needed.

## Conclusions

Most patients presenting with orbital floor fractures had satisfactory functional and visual results when titanium mesh was used to reconstruct the floor defect. Following surgery, notable improvement was seen in the restoration of extraocular muscle function, enophthalmos correction, and diplopia. Titanium mesh successfully maintained orbital volume, offered stable support for orbital content, and reduced postoperative complications. Its continuous use in the recent era as a dependable and biocompatible material for the repair of the orbital floor is supported by the results of this study. In setups like ours, due to resource-constrained conditions, orbital floor repair with titanium mesh is economical, especially for low-income patients. The minor complications can be managed without extra burden on the hospital and patients, as most of them are managed conservatively.
